# Associations between microvascular function and short-term exposure to traffic-related air pollution and particulate matter oxidative potential

**DOI:** 10.1186/s12940-016-0157-5

**Published:** 2016-07-26

**Authors:** Xian Zhang, Norbert Staimer, Tomas Tjoa, Daniel L. Gillen, James J. Schauer, Martin M. Shafer, Sina Hasheminassab, Payam Pakbin, John Longhurst, Constantinos Sioutas, Ralph J. Delfino

**Affiliations:** 1 Department of Epidemiology, School of Medicine, University of California, Irvine, Irvine 224 Irvine Hall, Irvine, CA 92617-7555 USA; 2 Department of Statistics, School of Information and Computer Sciences, University of California, Irvine, Irvine, CA USA; 3 Environmental Chemistry and Technology Program, University of Wisconsin-Madison, Madison, WI USA; 4 Department of Civil and Environmental Engineering, Viterbi School of Engineering, University of Southern California, Los Angeles, CA USA; 5 Susan Samueli Center for Integrative Medicine, and Cardiology Division, Department of Medicine, School of Medicine, University of California, Irvine, Irvine, CA USA

**Keywords:** Microvascular function, Air pollution, Oxidative potential, Particulate matter components

## Abstract

**Background:**

Short-term exposure to ambient air pollution has been associated with acute increases in cardiovascular hospitalization and mortality. However, causative chemical components and underlying pathophysiological mechanisms remain to be clarified. We hypothesized that endothelial dysfunction would be associated with mobile-source (traffic) air pollution and that pollutant components with higher oxidative potential to generate reactive oxygen species (ROS) would have stronger associations.

**Methods:**

We carried out a cohort panel study in 93 elderly non-smoking adults living in the Los Angeles metropolitan area, during July 2012-February 2014. Microvascular function, represented by reactive hyperemia index (RHI), was measured weekly for up to 12 weeks (*N* = 845). Air pollutant data included daily data from regional air-monitoring stations, five-day average PM chemical components and oxidative potential in three PM size-fractions, and weekly personal nitrogen oxides (NO_x_). Linear mixed-effect models estimated adjusted changes in microvascular function with exposure.

**Results:**

RHI was inversely associated with traffic-related pollutants such as ambient PM_2.5_ black carbon (BC), NO_x_, and carbon monoxide (CO). An interquartile range change increase (1.06 μg/m^3^) in 5-day average BC was associated with decreased RHI, −0.093 (95 % CI: −0.151, −0.035). RHI was inversely associated with other mobile-source components/tracers (polycyclic aromatic hydrocarbons, elemental carbon, and hopanes), and PM oxidative potential as quantified in two independent assays (dithiothreitol and *in vitro* macrophage ROS) in accumulation and ultrafine PM, and transition metals.

**Conclusions:**

Our findings suggest that short-term exposures to traffic-related air pollutants with high oxidative potential are major components contributing to microvascular dysfunction.

**Electronic supplementary material:**

The online version of this article (doi:10.1186/s12940-016-0157-5) contains supplementary material, which is available to authorized users.

## Background

Previous studies have reported positive associations of cardiovascular morbidity and mortality with short-term exposure to air pollutions [1–3]. While precise pathways underlying these associations have yet to be clarified, it has been hypothesized that the short-term cardiovascular effect of air pollution exposure may be mediated by induction of abnormal vascular responses characterized by reduced endothelium-mediated vasodilation and vessel constriction [2, 4]. Most previous studies have focused on macrovasculature endothelial function assessed using flow-mediated dilatation (FMD) of the brachial artery. However, emerging research shows that dysfunction in the microvascular circulation may be an important dimension of many cardiovascular conditions [5, 6]. A recent cross-sectional study investigated microvascular function using peripheral arterial tonometry and reported associations between baseline pulse amplitude and short-term exposure to ambient air pollutants, including particulate matter with aerodynamic diameter < 2.5 μm (PM_2.5_), black carbon (BC), and particle number concentrations, but not with vasodilator response [7]. A cohort panel study examined microvascular function in the retinal blood vessels and suggested that short-term exposure to higher levels of particulate matter with aerodynamic diameter < 10 μm (PM_10_) and BC may be associated with damage to the retinal microvasculature [8]. In a cross-over study among 53 healthy non-smoking women, UFP exposure was associated with a decrease in microvascular function that was measured by peripheral artery tonometry during physical activity [9]. To our knowledge, no cohort panel studies have evaluated relationships between peripheral microvascular function and air pollution, and there are no data on the importance of particle oxidative potential or specific particle components on microvascular function.

It is important to note in this regard that ambient air particles are a complex mixture of numerous components originating from different sources [1], each with complex particle size distributions. This may result in different adverse health effects. An increasing toxicology literature suggests that ultrafine particles (aerodynamic diameter smaller than approximately 0.1–0.2 μm) as compared with larger particles, which can dominate PM_2.5_ mass, may have greater adverse cardiovascular effects because of higher deposition efficiency and larger surface area [10], as well as higher redox activity [11]. In the present paper, we used repeated measurements to evaluate associations between microvascular function and size-fractionated PM (including their chemical composition and oxidative potential), in an elderly cohort living in the Los Angeles metropolitan area. Many previous epidemiological studies (especially time series of morbidity and mortality outcomes) have relied on uncharacterized PM_2.5_ and PM_10_ mass data, often from local regulatory agencies. In order to thoroughly characterize the exposure, our present study measured chemical composition in coarse (PM_2.5–10_), accumulation (PM_0.18–2.5_), and ultrafine (PM_0.18_) size fractions of PM [12, 13].

## Methods

### Study design

This study was a cohort panel design consisting of repeated measures of outcomes and exposures for 93 elderly non-smoking adults (age ≥ 65 years) living in the Los Angeles metropolitan area. Each subject effectively serves as his/her own control in this design. The elderly population is particularly susceptible to the adverse cardiovascular effects of air pollution exposure [1]. We followed subjects for up to 12 weeks, 6 weeks each during the warm season (July–October) and cool season (November–February) to incorporate seasonal differences in air pollution levels [14]. Exclusion criteria included: 1) employment outside of the monitored community (18 km radius); 2) smoking within the last 12 months; 3) exposure to environmental tobacco smoke at home or on a regular basis at other locations; 4) psychiatric disorder, dementia, alcohol or drug abuse; 5) dialysis treatment or renal failure; 6) daily oral corticosteroids; 7) active cancer; and 8) medical conditions that prevent the subject from giving blood or from performing the plethysmography procedure for measuring microvascular function. Observations following the previous 7 days when subjects reported any acute infection (8.16 %) were excluded *a priori* given their known major impacts on systemic inflammation.

### Outcome measurement

We collected background questionnaires at the beginning of the study that included medical history, socioeconomic status, medications, history of active smoking, and environmental exposure profile. Concurrently, a fasting blood sample was taken to obtain plasma lipid profiles and glucose levels.

At each follow-up visit, microvascular function (specifically arteriolar) was measured by forearm blood flow dilatation response to brachial artery occlusion using a noninvasive plethysmograph (EndoPAT 2000, Itamar Medical, Israel), yielding the reactive hyperemia index (RHI) score. A low RHI score indicates impaired endothelial function. The measurement protocol has been described previously [15]. Briefly, finger plethysmography was recorded on both arms in supine position in a quiet private room. Each measurement consists of a 5-min baseline measurement, a 5-min occlusion of the brachial artery (at least 60 mmHg above the systolic blood pressure) and a 5-min post-occlusion measurement (reactive-hyperemia response). Occlusion was performed on the non-dominant upper arm (test arm) and no occlusion was given on the dominant arm (control arm). RHI was the outcome variable, which was calculated as the increase in peripheral arterial tone signal amplitude (post-occlusion to pre-occlusion ratio). Systolic and diastolic blood pressures were measured before the EndoPAT using the Omron 7015IT (Omron Health Care, Kyoto, Japan) with direct computer linkage [16]. Due to space limitations in our clinics, our blood pressure measurements were taken under the non-standard conditions, namely insufficient time for subjects to rest (<5 min), as well as noisy and potentially stressful level of social activity in the common areas. Thesefore, blood pressure measurements were only used to assist in setting the cuff inflation pressure for the EndoPAT. Daily medication use and acute infectious disease status were ascertained by a patient self-report diary, filled out daily and collected at each weekly visit.

### Exposure measurements

Ambient air pollutants included hourly concentrations of U.S. Environmental Protection Agency criteria air pollutants, including PM_2.5_, carbon monoxide (CO), nitrogen oxides (NO_x_, NO+ NO_2_) and ozone (O_3_), and meteorological data including temperature and relative humidity. These data were obtained from the South Coast Air Quality Management District (SCAQMD) monitoring stations in the targeted study areas that included an approximate 18 km radius around the central air monitoring stations. Daily exposure data were calculated from the hourly data, when ≥ 75 % of daily data were available. Missing rates for daily PM_2.5_, CO, NO_x_, and O_3_ were 7.38 %, 6.83 %, 25.96 % and 9.02 %, respectively. Daily missing data for ambient air pollutants were imputed using regression modeling, predicted by the exposure data from the stations with non-missing data in the study area. A description of missing imputation methods is provided in Additional file 1: online supplement 1 and Table S1. Ambient air pollutant concentrations for 1-day, 3-day, 5-day and 7-day averages preceding clinic follow-ups were calculated from the daily data. This span of averaging times was representative of regression effect estimates for all averaging times across the previous week.

Our study team collected hourly PM_2.5_ BC (Aethalometer model AE22, Magee Scientific, Berkeley, CA) and 5-day integrated concentrations of PM_0.18_ (ultrafine mode), PM_0.18–2.5_ (accumulation mode) and PM_2.5–10_ (coarse mode) (MOUDI, model 100–1, MSP, Inc., Minneapolis, MN) at the University of Southern California (USC) monitoring sites. Five days of continuous particle collection before each clinic visit was necessary to obtain a sufficient amount of sample for the chemical and oxidative potential assays described below. The USC monitoring site for the first year of study was approximately 3 km southwest of the SCAQMD central air monitoring station where criteria air pollutants were measured in downtown Los Angeles (see Additional file 1: Figure S1). Our USC site for the second year of study was at the same location as the SCAQMD station in Anaheim. Following gravimetric measurements using a highly precise (±0.001 mg) microbalance (Mettler Toledo Inc., Columbus, OH, USA), filters were sectioned and disturbed for PM chemical characterization. To quantify the elemental and organic carbon (EC and OC, respectively) content of the samples, a 1.5 cm^2^ punch of the quartz/aluminum filters was analyzed by National Institute for Occupational Safety and Health Thermal Optical Transmission method [17]. Speciated organics, including total polycyclic hydrocarbons (PAHs), hopanes, and organic acid (see Additional file 1: Table S2) were measured using gas chromatography mass spectrometry [18]. Total elemental composition of the samples was measured by digestion of a section of the Teflon filter-collected PM using a microwave added, sealed bomb, mixed acid digestion. Digests were subsequently analyzed by high-resolution inductively coupled plasma sector field mass spectrometry (SF-ICPMS). The present study focuses on the measured transition metals (V, Cr, Mn, Ni, Cu, and Fe) because of their potential to induce oxidative stress through Fenton reactions.

*In vitro* redox activity of PM was measured by two different methods: alveolar macrophage reactive oxygen species (ROS) assay [19], representing the biotic oxidative potential of particle mixtures, and dithiothreitol (DTT) activity [20], representing capacity to generate abiotic chemically-produced ROS. Biotic ROS production was quantified by extracting the filter with 1.00 ml of Milli-Q water. We then exposed rat alveolar macrophage cells (NR8383, American Type Culture Collection) and the fluorescent probe DCFH-DA in 96-well plates to both unfiltered (total ROS) and filtered (water-soluble ROS) (0.22 μm polypropylene syringe filter) PM extracts. Fluorescence intensity then was measured using a plate reader and represents the cell-based oxidative generating capacity of PM. A model of microbial particles, un-opsonized Zymosan (a β-1,3-polysachharide of D-glucose) served as a positive ROS control as it binds to Toll-like receptor-2 on macrophage cells and then activates a strong respiratory burst and ROS production. ROS results are reported in μg Zymosan equivalent units per m^3^ air based the product of μg Zymosan equivalents/μg sample times the μg PM per m^3^ air. Details of the macrophage ROS method are described in detail elsewhere [19]. DTT activity was quantified using a well-established method [21] on extracts of 5-day composites of the three different size-fractionated PM from quartz filters. DTT activity represents the ability of the PM extract to catalyze electron transfer from DTT to oxygen, thereby generating superoxide radicals.

Seven-day average personal exposures to NO_x_ were collected using the Ogawa passive badge sampler (Ogawa & Co. USA, Inc. Pompano Beach, FL). Subjects were instructed to wear the sampler clipped outside of clothing and placed near the bedside. Personal NO_x_ was collected on cellulose fiber filters and concentrations were determined by a spectrophotometer at a wavelength of 545 nm in our laboratory following manufacturer’s specification [22]. To capture the combined health-related effects of temperature and relative humidity, we calculated hourly heat index using methods developed in a previous study [23].

### Statistical analysis

To account for the clustering of longitudinal repeated measurements taken on each subject, we performed repeated measurement analysis to investigate the association between RHI and air pollutants using a linear mixed effects model that included a random subject intercept to account for the correlation of repeated measures. The general form of this model is given by:

$$ {Y}_{i,j}={a}_{i,}+\alpha {Z}_{i,}+\beta {X}_{i,j}+\gamma {W}_{i,j}+{\varepsilon}_{i,j} $$

Here, *i* indexes subject (i =1,…, 93), *j* indexes outcome measurement on each subject (*j* =1,…,12). *Y*_*i,j,*_ is the outcome measurement, *a*_*i*_ is the random subject intercept, *Z*_*i*_ is a vector of time-independent subject characteristics (e.g. sex or medical conditions), *X*_*i,j*_ is a vector of time-dependent air pollutant exposure levels (of primary interest), *W*_*i,j*_ is a vector of time-dependent covariates (time trend, medications, and weather), and *ε*_*i,j*_ denotes random within-person error in the outcome measurement. The best covariance structure of *ε*_*i,j*_ was selected using Akaike’s information criterion (AIC) and was the autoregressive moving average (ARMA) (1,1) covariance structure. To compare associations across different pollutants, the magnitude of effect was expressed relative to an interquartile range (25th to 75th percentile) difference in each pollutant concentrations. All statistical analyses were performed using R version 3.0.3 (R Foundation for Statistical Computing, Vienna, Austria) or SAS 9.3 software (SAS, Cary, NC).

Time invariant subject characteristics were controlled by study design and the specified repeated measures model, and hence were not included as adjustment covariates. Heat index was inversely associated with RHI, albeit nonsignificant. An *a priori* covariate was heat index with the same lag as the pollutant to adjust for the effects of weather. Other time-dependent potential confounders were tested in the models. Exercise [24] and/or food intake [25] can change peripheral blood flow and potentially affect microvascular function. In the model, we adjusted exercise and/or food intake within an hour before subjects came to the clinic as a potential confounding factor for microvascular function. Days of gas stove use per week were significantly associated with increased personal NO_x_ and were adjusted in the models of personal NO_x_ because the exposure of interest was outdoor fossil fuel sources of NO_x_. Long-term temporal trend was tested by including cubic splines using different knots for day of study. However, it did not significantly change the estimation or improve model fit. As a result, adjustment for temporal trend was not included in our final model.

The impact of influential observations was assessed using the Cook’s D statistic and standardized residual diagnostics, at both the individual observations level and clustered subjects level [26]. No evidence to suggest a departure from normality was observed and no significant influential observations were detected.

Risk factors for cardiovascular disease may be potential effect modifiers of the association between microvascular function and air pollution. Accordingly we tested them by incorporating multiplicative interactions with air pollutants in exploratory analyses. These risk factors included age (>75 years old), sex, obesity (body mass index: BMI ≥ 30 kg weight/m^2^ height), measured hypertension (systolic blood pressure > 140 or diastolic blood pressure > 90), diabetes mellitus, hypercholesterolemia by history, high cholesterol (total cholesterol > 200 mg/dL), high low-density lipoprotein (LDL) concentration (≥140 mg/dL), total cholesterol/high-density lipoprotein (HDL) > 3.5, history of cardiovascular disease, and former smokers. We also tested differences in association between the cohort in Los Angeles and the cohort in Anaheim. Evidence of significant interaction was considered at a nominal product term *p*-value < 0.1 to avoid increased type II errors in these hypothesis-generating analyses.

Several sensitivity analyses were conducted. First, to investigate potential exposure error, we restricted the analysis to the subjects who lived within the 90^th^ percentile of subjects’ residential distance to the stations (11.3 km for the SCAQMD monitoring stations and 13.1 km for the USC monitoring sites). Second, we limited the analysis to measured ambient exposure, rather than exposure including imputed data from the next nearest station. Third, we excluded days with extreme heat index (<52.5 and > 75.04, 10^th^ percentile and 90^th^ percentile of the heat index during the study period, respectively) because the decreased ventilation at subjects’ residential buildings during those days may lead to an increased exposure error. Finally, we tested for the independent effects of photochemically-related and primary air pollutants by using two-pollutant models with O_3_ and another primary pollutant.

## Results

Detailed information on the characteristics of study subjects are listed in Table 1. The subjects were 65 to 96 years old, and two-thirds of the subjects were female. All were currently non-smokers, though approximately 40 % were former smokers. Among the 93 subjects, there are 59 non-Hispanic Whites, 9 Hispanics, 11 African Americans, 9 Asians and 5 other race/ethnicities. Approximately one-third of subjects were obese (BMI ≥ 30 kg/m^2^) and one-third were overweight (25 kg/m^2^ ≤ BMI < 30 kg/m^2^). More than 60 % of subjects had history of a hypertension, and over half had a history of dyslipidemia. Sixteen percent had diabetes. The mean RHI score was 1.94, which was in the normal endothelial function range (>1.67) according to the EndoPAT manufacturer’s manual [27].

**Table 1:** Characteristics of subjects (*N* = 93)

Characteristic	Mean ± SD or N(%)
Age (years) ± SD	74.9 ± 7.6
Body mass index (kg/m^2^) ± SD	27.8 ± 5.5
Overweight (25–29.9)	35 (37.6)
Obesity (≥30)	28 (30.1)
Male	25 (26.9)
Former smoker	39 (41.9)
Reactive hyperemia index (RHI) score	1.95 (0.4)
Cardiovascular history	
Coronary artery disease	16 (17.2)
Congestive heart failure	8 (8.6)
Stroke	9 (9.7)
Hypertension	62 (66.7)
Hypercholesterolemia (by history)	50 (53.8)
Lipid Profile	
Total cholesterol > 200 mg/dL	37 (39.8)
LDL-C > 130 mg/dL	29 (31.2)
HDL-C < 50 mg/dL for women; < 40 mg/dL for men	25 (26.9)
Adult-onset diabetes mellitus	21 (22.6)
Medications:	
Anti-hypertensive medications	62 (66.7)
HMG-CoA reductase inhibitors (statins)	45 (45.2)

Table 2 provides metrics for the 24h-average concentrations of ambient air pollutants, 7-day average personal NO_x_, and 5-day average size-fractionated PM air pollutants. Data for specific transition metals is presented in the Additional file 1: Table S3. For 24h ambient pollutants at the central sites, NO_x_ had the highest missing rates (29.95 %), and all other pollutants had less than 10 % of missing data. Personal NO_x_ had 15.91 % missing data due to measurement errors where NO_x_ concentration was less than NO_2_, subject noncompliance, or incorrect sampling times. For size-fractionated PM components, up to 4 of the planned 48 weeks were missing due to equipment failure or power outages at the Anaheim site. Ninety-seven percent of the study period had average 24h PM_2.5_ below the National Air Quality Standard recommended upper limit of 35 μg/m^3^. Total PAHs and hopanes were higher in PM_0.18–2.5_ than in PM_0.18_. Both DTT and ROS were highest in PM_0.18–2.5_, and were higher in PM_2.5–10_ than in PM_0.18_. For most transition metals (V, Cr, Mn, Cu and Fe), the highest mass concentrations were observed in PM_2.5–10_ (Additional file 1: Table S3).

**Table 2:** Descriptive statistics of air pollutant measurements

	N (Missing)	Mean (SD)	IQR	Min	Max
Personal Exposures (7-day average)					
NO_x_ (ppb)	729 (116)	28.07 (19.66)	20.87	2.21	160.13
Ambient Exposures (24h Averages)					
Black Carbon (μg/m^3^)	320 (16)	1.28 (0.85)	1.06	0.21	5.21
PM_2.5_ (μg/m^3^)	309 (27)	17.56 (7.70)	9.62	3.83	49.23
CO (ppm)	311 (25)	0.54 (0.24)	0.32	0.11	1.59
Ozone (ppb)	303 (33)	23.16 (9.25)	12.66	1.33	48.51
NO_x_ (ppb)	241 (95)	35.42 (27.69)	31.02	3.6	175.63
Heat Index (F°)	366 (0)	66.09 (8.39)	12.56	42.57	84.33
Size-fractionated PM (5-day average)					
Mass (μg/m^3^)					
PM_0.18_	45 (3)	2.41 (0.86)	1.13	1.17	4.81
PM_0.18_ – PM_2.5_	45 (3)	8.64 (3.21)	4.01	4.40	19.43
PM_2.5_ – PM_10_	44 (4)	14.91 (6.78)	7.80	4.45	35.36
Total PAH (ng/m^3^)					
PM_0.18_	45 (3)	0.32 (0.22)	0.30	0.03	0.77
PM_0.18_ – PM_2.5_	45 (3)	0.46 (0.45)	0.46	0.21	1.98
Hopanes (ng/m^3^)					
PM_0.18_	45 (3)	0.17 (0.12)	0.19	0.01	0.44
PM_0.18_ – PM_2.5_	45 (3)	0.21 (0.20)	0.21	0.00	0.82
OA (μg/m^3^)					
PM_0.18_	45 (3)	26.21 (10.84)	14.45	9.64	57.98
PM_0.18_ – PM_2.5_	45 (3)	18.12 (13.99)	16.76	0.56	61.43
OC (μg/m^3^)					
PM_0.18_	45 (3)	1.18 (0.39)	0.42	0.47	2.35
PM_0.18_ – PM_2.5_	45 (3)	1.54 (0.78)	1.11	0.53	3.48
PM_2.5_ – PM_10_	45 (3)	0.67 (0.22)	0.30	0.28	1.12
EC (μg/m^3^)					
PM_0.18_	45 (3)	0.26 (0.13)	0.16	0.09	0.60
PM_0.18_ – PM_2.5_	45 (3)	0.15 (0.12)	0.18	0.01	0.45
PM_2.5_ – PM_10_	45 (3)	0.04 (0.03)	0.05	0.00	0.09
Total ROS (μg Zym/m^3^)^a^					
PM_0.18_	45 (3)	19.8 (12.21)	16.60	2.10	53.20
PM_0.18_ – PM_2.5_	45 (3)	136.69 (94.62)	125.90	26.20	394.00
PM_2.5_ – PM_10_	44 (4)	60.19 (49.73)	76.45	8.70	181.00
Water-soluble ROS (μg Zym/m^3^)					
PM_0.18_	45 (3)	16.74 (11.49)	13.60	1.50	50.60
PM_0.18_ – PM_2.5_	45 (3)	117.05 (87.66)	95.60	18.60	421.90
PM_2.5_ – PM_10_	44 (4)	25.04 (22.63)	34.05	2.40	84.20
Dithiothreitol (nmol/min/m^3^)					
PM_0.18_	45 (3)	0.10 (0.05)	0.06	0.02	0.24
PM_0.18_ – PM_2.5_	45 (3)	0.26 (0.08)	0.08	0.12	0.48
PM_2.5_ – PM_10_	44 (4)	0.24 (0.11)	0.14	0.10	0.53
Transition metals^b^ (ng/m^3^)					
PM_0.18_	45 (3)	49.20 (40.43)	56.2438	7.22	153.39
PM_0.18_ – PM_2.5_	45 (3)	72.02 (52.86)	46.0233	12.89	237.11
PM_2.5_ – PM_10_	44 (4)	396.57 (171.18)	219.9526	111.97	914.57

*Abbreviations*: *IQR* interquartile range, *CO* carbon monoxide, *PM* particulate matter, *PAHs* polycyclic aromatic hydrocarbons, *OC* organic carbon, *EC* elemental carbon, *ROS* Reactive oxygen species

^a^Zym: μg Zymosan equivalent units

^b^Total sum of transition metals include V, Cr, Mn, Ni, Cu and Fe

As expected, concentrations of traffic-related air pollutants (BC, NO_x_, CO, EC, PAHs, and hopanes) were higher in the more densely urban Los Angeles region than in the more suburban Anaheim (Additional file 1: Table S4). The correlation between 5-day averages of PM_2.5_ BC and EC were much lower in Los Angeles (R = 0.54) than in Anaheim (R = 0.93). This is likely because the sampling sites were different in Los Angeles but were the same in Anaheim (Additional file 1: Figure S1). The correlation between personal NO_x_ and ambient NO_x_, BC and CO was much stronger in Anaheim than in Los Angeles (Additional file 1: Table S5). This could be because of a greater influence of micro-environmental exposures, including local traffic, in Los Angeles than in Anaheim. Given these regional differences, we present correlations for combined regions in Tables 3 and 4 after mean-centering exposures by region. Spearman correlations of ambient air pollutants showed strong positive correlations among the traffic-related air pollutants (BC, NO_x_, CO, R > 0.87) (Table 3). These pollutants correlated weakly to PM_2.5_, especially in Los Angeles (Additional file 1: Table S5). Moderate to strong inverse correlations were observed for traffic-related air pollutants and O_3_, with stronger correlations in Anaheim than Los Angeles. For 5-day size-fractionated PM, total mass in PM_0.18_ was not correlated with total mass in PM_0.18–2.5_ and inversely correlated with total mass in PM_2.5–10_ (Table 4). Total PAHs and hopanes were strongly correlated (R = 0.89-0.91), suggesting that the primary source of PAHs was traffic since hopanes are unique tracers of vehicular emissions in the Los Angeles basin and found in the lubricant oils of diesel and gasoline vehicles [28]. Correlations between total PAHs and OC were strong in both PM_0.18_ and PM_0.18–2.5_ while the correlations between total PAHs and EC were stronger in PM_0.18_ than in PM_0.18–2.5_. Correlation between DTT and OC/EC was strong in PM_0.18_, and weak in PM_0.18–2.5_ and PM_2.5–10_, suggesting that oxidative potential may have different chemical determinants in these size fractions. Transition metals (except for V) were strongly correlated with OC/EC, total ROS and DTT in PM_2.5–10_ (R = 0.59-0.72) (Additional file 1: Table S6).

**Table 3:** Spearman correlation matrix of ambient, personal air pollutants and heat index^a^

	BC	CO	NO_x_	PM_2.5_	O_3_	Heat Index (F°)
24h averages of ambient exposures						
BC (μg/m^3^)		0.87	0.90	0.33	−0.69	−0.27
CO (ppm)			0.90	0.27	−0.73	−0.32
NO_x_ (ppb)				0.20	−0.77	−0.32
PM_2.5_ (μg/m^3^)					−0.06	0.09
O_3_ (ppb)						0.54
7-day averages of personal exposure^b^						
Personal NO_x_ (ppb)	0.47	0.45	0.47	0.16	−0.43	−0.25

^a^Pollutants are mean-centered by region

^b^Correlations for personal NO_x_ were calculated with 7-day average of ambient pollutants and heat index

**Table 4:** Spearman correlation matrix of 5-day average of components in size-fractionated PM^a^

	PM_0.18_									PM_0.18–2.5_										PM_2.5–10_						
Pollutants	T PAHs	Hopa-nes	OA	OC	EC	T ROS	WS ROS	DTT	T Metals	T mass	T PAHs	Hopa-nes	OA	OC	EC	T ROS	WS ROS	DTT	T Metals	T mass	OC	EC	T ROS	WS ROS	DTT	T Metals
PM_0.18_																										
Mass	0.58	**0.63**	0.29	**0.74**	**0.62**	0.47	0.45	**0.72**	**0.70**	0.00	**0.50**	0.44	0.24	0.52	0.24	0.18	0.14	0.38	0.57	−0.32	0.26	0.11	−0.40	−0.36	0.34	0.23
T PAHs		**0.89**	0.51	**0.80**	**0.75**	0.10	0.11	**0.76**	**0.81**	0.13	**0.94**	**0.86**	**0.71**	**0.87**	0.37	0.18	0.21	0.52	**0.68**	−0.60	0.55	0.51	−0.30	−0.42	0.57	**0.61**
Hopanes			**0.60**	**0.82**	**0.72**	0.17	0.16	**0.79**	**0.78**	0.13	**0.85**	**0.78**	0.58	**0.80**	0.42	0.16	0.20	0.56	**0.68**	−0.47	0.49	0.46	−0.27	−0.38	0.58	**0.61**
OA				0.52	0.27	0.09	0.11	0.42	0.30	0.21	0.54	**0.65**	0.55	0.54	0.25	0.08	0.23	0.45	0.37	−0.33	0.32	0.40	−0.10	−0.15	**0.66**	0.35
OC					**0.83**	0.45	0.44	**0.86**	**0.80**	0.08	**0.73**	**0.72**	0.43	**0.76**	0.46	0.26	0.28	**0.61**	**0.70**	−0.36	0.53	0.36	−0.25	−0.29	0.49	0.57
EC						0.42	0.42	**0.77**	**0.68**	0.19	**0.64**	0.51	0.36	**0.76**	0.58	0.39	0.28	0.57	**0.66**	−0.23	0.57	0.31	−0.07	−0.07	0.29	**0.67**
T ROS							**0.98**	0.41	0.25	0.01	−0.02	−0.02	−0.15	0.12	0.34	0.30	0.28	0.17	0.18	0.28	0.20	−0.04	0.12	0.13	−0.01	0.09
WS ROS								0.42	0.23	−0.01	−0.01	0.00	−0.12	0.13	0.35	0.28	0.27	0.15	0.16	0.26	0.21	0.00	0.12	0.15	0.00	0.11
DTT									**0.81**	0.00	**0.68**	0.59	0.30	**0.63**	0.50	0.14	0.17	0.44	**0.68**	−0.42	0.45	0.25	−0.27	−0.37	0.40	0.45
T Metals^b^										0.00	**0.76**	**0.61**	0.33	**0.64**	0.32	0.13	0.18	0.48	**0.80**	−0.46	0.50	0.33	−0.40	−0.51	0.42	0.51
PM_0.18–2.5_																										
Mass											0.18	0.12	0.21	0.36	0.19	**0.65**	**0.67**	0.48	0.24	0.26	0.15	0.29	0.47	0.43	0.32	0.24
T PAHs												**0.90**	**0.73**	**0.85**	0.32	0.09	0.22	0.49	**0.63**	−0.54	**0.60**	0.56	−0.29	−0.44	**0.67**	**0.65**
Hopanes													**0.77**	**0.81**	0.23	0.08	0.18	0.47	0.52	−0.60	0.55	0.51	−0.34	−0.43	**0.71**	0.52
OA														**0.70**	0.15	−0.03	0.14	0.35	0.27	−0.53	0.31	0.47	−0.20	−0.30	**0.61**	0.37
OC															0.47	0.31	0.35	**0.62**	**0.63**	−0.42	0.59	0.56	−0.09	−0.16	**0.63**	**0.67**
EC																0.41	0.46	0.25	0.27	0.15	0.31	0.33	0.27	0.29	0.18	0.52
T ROS																	**0.84**	0.37	0.30	0.27	0.11	0.14	0.33	0.47	0.11	0.14
WS ROS																		0.40	0.32	0.22	0.16	0.26	0.43	0.44	0.30	0.25
DTT																			0.57	−0.16	0.42	0.35	0.14	0.01	0.41	0.53
T Metals^b^																				−0.37	0.51	0.23	−0.11	−0.20	0.32	0.53
PM_2.5–10_																										
Mass																					−0.04	−0.01	**0.60**	**0.67**	−0.31	−0.06
OC																						0.55	0.06	0.00	0.32	0.75
EC																							0.19	0.11	0.39	0.51
T ROS																								**0.87**	−0.11	0.23
WS ROS																									−0.20	0.09
DTT																										0.39

*Abbreviations*: *DTT* dithiothreitol, *EC* elemental carbon, *OA* Organic acids, *OC* organic carbon, *PM* particulate matter, *ROS* reactive oxygen species, *T* total, *WS* water-soluble

Bold numbers indicate correlation values ≥ 0.60 and *P* < 0.05

^a^Pollutants are mean-centered by region

^b^Total sum of transition metals include V, Cr, Mn, Ni, Cu and Fe

**Fig. 1:**
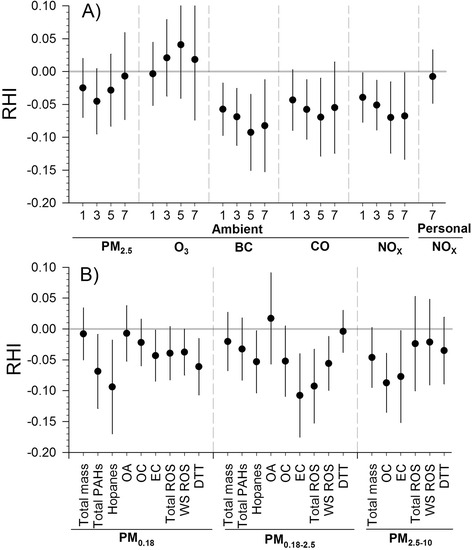
Association of microvascular function with a one interquartile range increase of ambient and personal pollutants. Exposures were averaged across 1 day, 3 days, 5 days, and 7 days preceding each subject’s reactive hyperemia index (RHI) measurement (**a**) and the PM components in three different size-fractions for exposures averaged across 5 days preceding each subject’s RHI measurement (**b**). BC: black carbon; DTT: dithiothreitol; EC: elemental carbon; OA: Organic acids; OC: organic carbon; PAHs: polycyclic aromatic hydrocarbons; PM particulate matter; ROS: reactive oxygen species; WS: water-soluble

Figure 1 presents the relationships between RHI, as a measure of microvascular function, and interquartile range increases in ambient air pollutants measured daily and 5-day size-fractionated PM components. RHI was inversely associated with ambient BC and NO_x_ for 1- through 7-day averaging times, and with CO for 3- and 5-day averages, indicating impaired endothelial function with exposure to these pollutants. RHI was not significantly associated with ambient PM_2.5_ or O_3_ at any averaging time, or with 7-day average personal NO_x_ (Fig. 1a). The strongest associations with ambient daily exposures were observed for 5-day averages. For example, an interquartile range increase in 5-day average BC (1.06 μg/m^3^) was associated with an RHI decrease of −0.093 (95 % confidence intervals, CI: −0.151, −0.035). We observed no significant regional differences of the association between RHI and ambient exposures, except for PM_2.5_ at 7-day average (product term *p* = 0.08), with a positive association in Los Angeles and a negative association in Anaheim (95 % CI; for both regional associations were wide and contained 0.00) (Additional file 1: Figure S2).

**Fig. 2:**
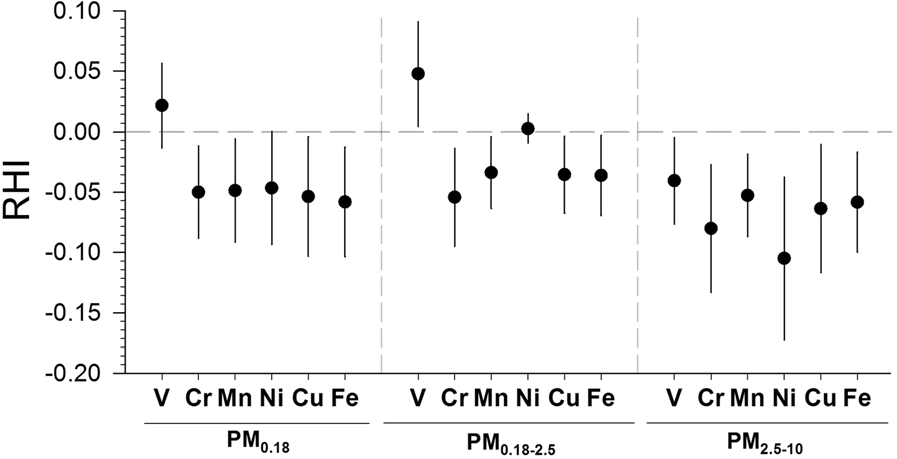
Association of microvascular function with a one interquartile range increase of selected transition metals. Transition metals were measured in three size-fractions for exposures averages across 5 days preceding each subject’s reactive hyperemia index (RHI) measurement

RHI was not significantly associated with 5-day total mass of PM_0.18_ or PM_0.18–2.5_, but was marginally associated with total mass in PM_2.5–10_ (−0.046, 95 % CI: −0.095, 0.004; Fig. 1b). We did observe that RHI was inversely associated with total PAHs, hopanes and DTT in PM_0.18_, marginally inversely associated with PM_0.18_ EC, total and water-soluble ROS, but not PM_0.18_ organic acids (OA) or OC (representing a mixture of primary and secondary organic aerosols). RHI was associated more strongly (inversely) with EC, total and water-soluble ROS in PM_0.18–2.5_ than in the other particle sizes. RHI also was inversely associated with OC in PM_2.5–10_ (a minor fraction of that particle size), but not in the other particle sizes, suggesting that the fraction of primary and secondary OC may be different in PM_2.5–10_ or that OC is correlated with other components (e.g. metals) associated with RHI (Additional file1: Table S6). We found that RHI was inversely associated with all transition metals in PM_2.5-10, _with the transition metals Cr, Mn, Ni, Cu and Fe but not Ni or V in PM_0.18–2.5_ and with the transition metals Cr, Mn, Ni, Cu, and Ni but not V in PM_0.18_. (Fig. 2).

**Fig. 3:**
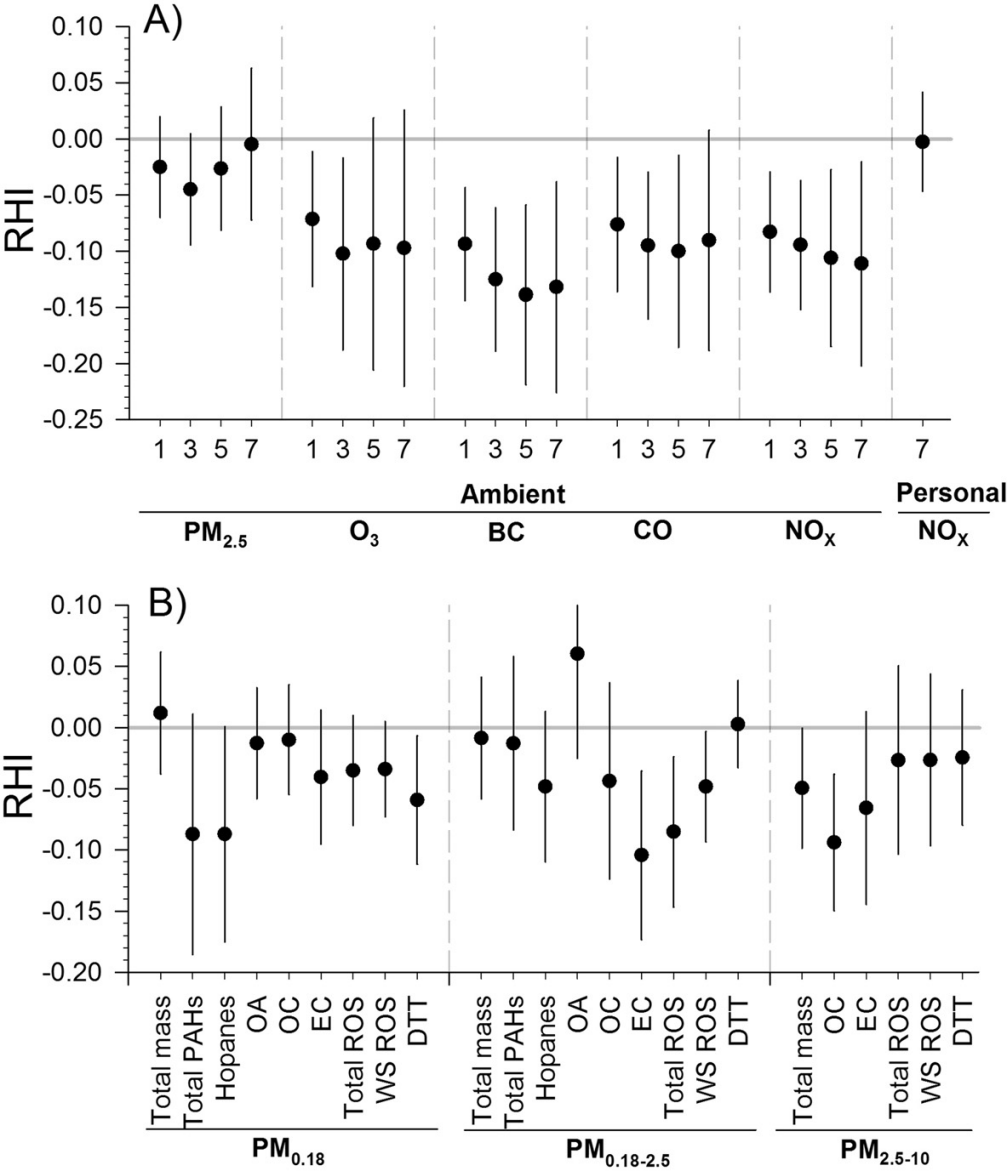
Sensitivity analysis of relations between microvascular function and air pollution: two-pollutant models. Association of reactive hyperemia index (RHI) with a one interquartile range increase of ambient and personal air pollutants for exposures averaged across 1 day, 3 days, 5 days, and 7 days preceding each subject’s measurement (**a**) and the PM components in three different size-fractions for exposures averaged across 5 days preceding each subject’s RHI measurement (**b**). The sensitivity analysis is adjusting for ozone with the same averaging time, except for the ozone model, which adjusts for black carbon with the same averaging time. BC: black carbon; DTT: dithiothreitol; EC: elemental carbon; OA: Organic acids; OC: organic carbon; PAHs: polycyclic aromatic hydrocarbons;  PM particulate matter; ROS: reactive oxygen species; WS: water-soluble

For two-pollutant models, the magnitude of the nonsignificant associations for PM_2.5_ and personal NO_x_ were largely unchanged with O_3_ in the model (Fig. 3a). Estimations of association for ambient BC, CO, NO_x_ showed even greater decreases in microvascular function by 45–113 % in RHI after adjusting for O_3_ than in single-pollutant models (Fig. 1a), although the confidence intervals were wider. We also noted that adjusting for BC in the model with O_3_, the estimations of association for O_3_ changed from positive in single-pollutant models (Fig. 1a) to negative in two-pollutant models (Fig. 3a), and became significant for 1- and 3-day averages. To address the possibility of an interaction between primary and secondary air pollutants (BC and O_3_, respectively), we further tested a model with the product term of BC and O_3_ and found significant positive interactions (*p* <0.1) of BC and O_3_ on RHI at 3-day and 5-day averages, suggesting synergism (Additional file 1: Figure S3). The estimates of association for 5-day size-fractionated PM and components remained relatively unchanged after adjusting for O_3_ (Fig. 3b).

Associations also persisted when we restricted the analysis to subjects living within the 90^th^ percentile of all subjects’ residential distance to monitoring stations (Additional file 1: Figure S4). When we excluded imputed ambient exposure data, the associations for PM_2.5_, O_3_ and BC remained similar. Conversely these associations became slightly weaker for NO_x_ and CO (Additional file 1: Figure S5). We did not observe significant changes when we excluded days with extreme heat index (data not shown).

**Fig. 4:**
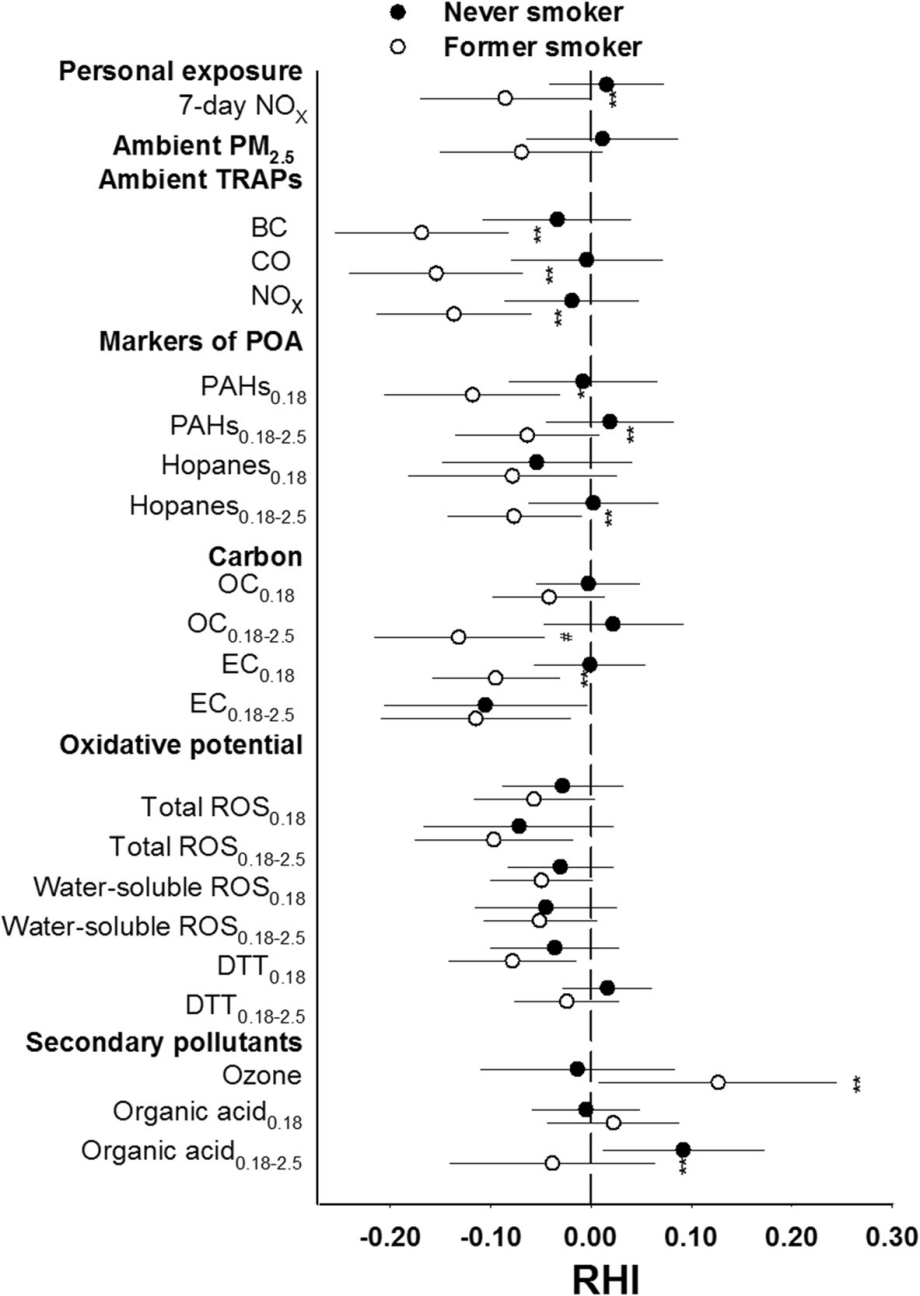
Effect modification of relations between microvascular function and air pollution by never and former smoker. Association of reactive hyperemia index (RHI) with one interquartile increase of selected air pollutant exposures averaged across 5 days preceding each subject’s RHI measurement. *p < 0.1, **p < 0.05, ^#^ *p* < 0.01, compared with no effect modification by smoking status. BC: black carbon; DTT: dithiothreitol; EC: elemental carbon; OC: organic carbon; PAHs: polycyclic aromatic hydrocarbons;  PM particulate matter; ROS: reactive oxygen species

**Fig. 5:**
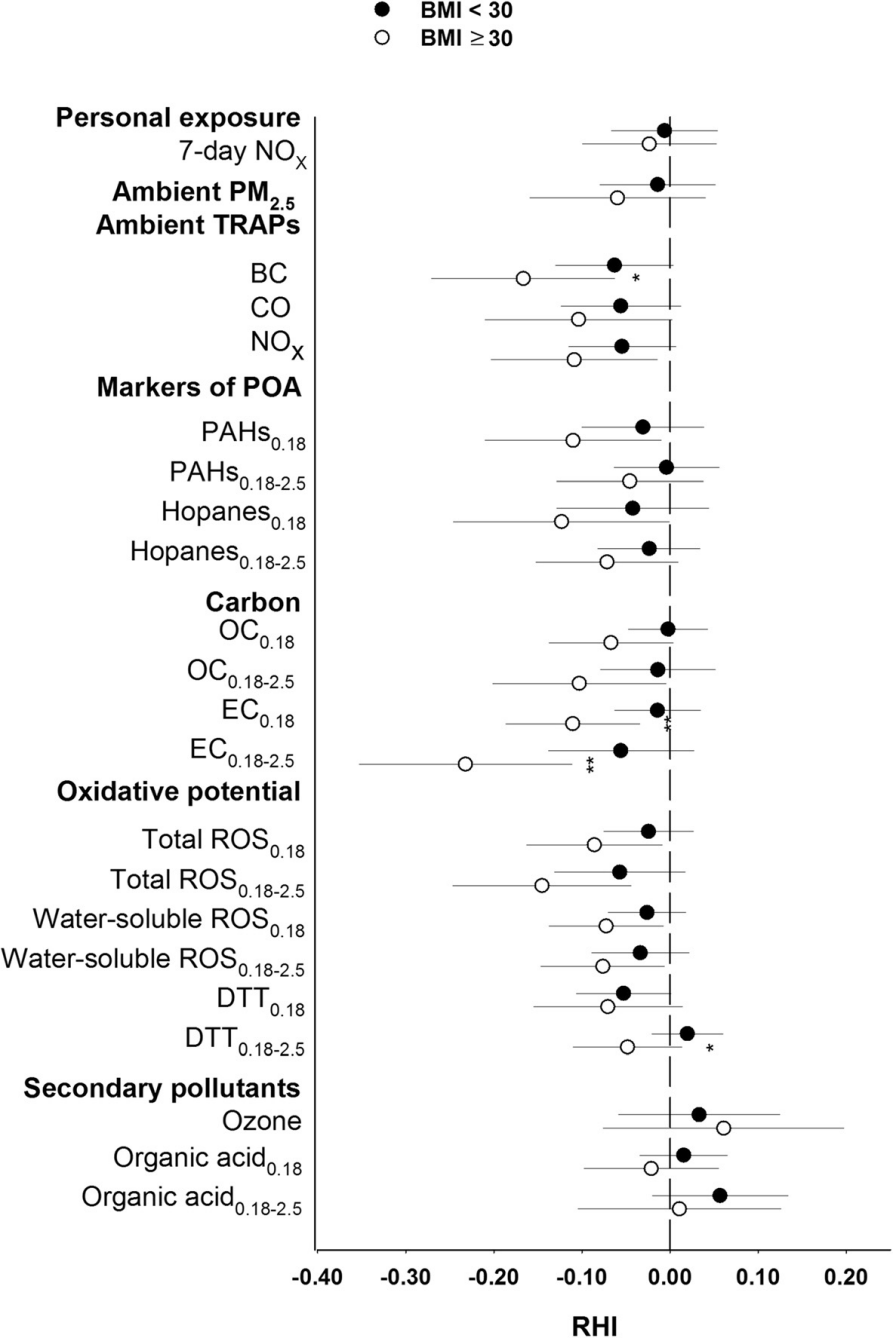
Effect modification of relations between microvascular function and air pollution by obesity status. Association of RHI score with one interquartile range increase of selected air pollutant exposures averaged across 5 days preceding each subject’s reactive hyperemia index (RHI) measurement: *p < 0.1, **p < 0.05, compared with no effect modification by obesity status. BC: black carbon; BMI: body mass index; DTT: dithiothreitol; EC: elemental carbon; OC: organic carbon; PAHs: polycyclic aromatic hydrocarbons; PM particulate matter; ROS: reactive oxygen species

We evaluated effect modification of associations between RHI and 5-day average air pollutants (except for personal NO_x_ since only 7-day averages were available). Five-day averages were selected because we found that the largest and most consistent associations with RHI were for 5-day averages. Also, this is the averaging time for the PM components. We observed evidence of effect modification by smoking status on the association between RHI and exposures to traffic-related air pollutants (ambient BC, CO, NO_x_, personal NO_x_, PAHs, hopanes, EC) with stronger inverse associations estimated for former smokers than for individuals who never smoked (Fig. 4). Subjects who were obese generally had stronger inverse associations of RHI with exposure to air pollutants, except for secondary pollutants (Fig. 5). Significant modifying effects (*p* < 0.1) were observed for exposures to ambient PM_2.5_ BC, and EC in PM_0.18_ and PM_0.18–2.5_. We observed no evidence of statistically significant interaction between exposures and age, sex, diabetes status, lipid factors, hypertension or history of cardiovascular diseases (data not shown).

## Discussion

We found decreased microvascular endothelial function in relation to increases in short-term exposure to air pollution. These associations were observed primarily for markers of primary fossil fuel combustion sources (EC, BC, CO, NO_x_, PAHs, and hopanes). One of the strongest associations was observed for 5-day average BC with an interquartile range increase associated with a RHI decrease of −0.093. There is no standardized guideline for the clinical relevance of changes in RHI. However, previous studies indicate that a low RHI score is independently associated with adverse cardiovascular outcomes. For example, the hazard ratio for cardiovascular events per 1.1 increase of RHI showed a significant decrease in risk 0.761 (CI: 0.691, 0.832) after controlling for other risk factors in a high risk cohort of 528 subjects [6].

We used hourly ambient air monitoring data over the seven days preceding outcome measurements and found that the strongest associations were for 5-day averages. Several controlled exposure studies have found that diesel exhaust is associated with impaired vascular function from as early as 2 h after the exposure and up to 24 h [29, 30]. Comparisons with the present study are difficult because previous studies used experimental study designs incorporating higher exposure levels (i.e. 300 μg/m^3^ particulate mass concentrations).

We found stronger associations of RHI with PAHs and hopanes in PM_0.18_ than in the PM_0.18–2.5_, which includes larger particles. In the Los Angeles metropolitan area, most outdoor PAHs in PM_0.18_ are expected to be from mobile sources [31]. The strong correlation of PAHs (R = 0.89) with hopanes (source markers of vehicular emissions) is consistent with this expectation. PAHs were more strongly correlated with DTT in PM_0.18_ than in PM_0.18–2.5_ indicating that PAHs in PM_0.18_ were more redox active. This is consistent with our findings that even though the concentration of PAHs and hopanes were higher in PM_0.18–2.5_ than in PM_0.18_ (Table 2), we observed stronger associations of RHI with PAHs and hopanes in PM_0.18_. The spatial variation in UFP is much greater than larger particle size fractions. Therefore, exposure error is expected to be much greater when regional monitoring data are used and this complicates direct comparisons between size fractions [32, 33]. Our previous panel study did not have PM_0.18_ components but did find that circulating biomarkers of inflammation were associated with PAHs and hopanes measured in quasi-ultrafine PM_0.25_ [34]. The biomarkers were not associated with other organic components or transition metals in the PM_0.25_. Furthermore, PAHs confounded nominal associations of biomarkers with PM_0.25_ mass. In a follow-up panel study with organic components also measured in the accumulation mode size fraction (PM_0.25–2.5_), we found that exposure markers of combustion-related air pollutants including PM_0.25–2.5_ PAHs and/or PM_0.25_ PAHs were positively associated with expression of genes in inflammatory and oxidative stress pathways, including *NFE2L2, Nrf2*-mediated genes (*HMOX1, NQO1*, and *SOD2*), *CYP1B1, IL1B*, and *SELP* [35]. In toxicological studies, it has been demonstrated that ultrafine particles have high levels of organic compounds and metals, and were more capable of generating ROS [36] and pro-inflammatory responses [37].

This is the first epidemiological study reporting a decrease in microvascular function in relation to markers of PM oxidative potential. This is consistent with our previous novel findings for associations with chemical components such as PAHs with known pro-oxidant effects in cell cultures [38]. As noted by Higashi et al. [39], the underlying mechanisms may be increased production of ROS during oxidative stress that inactivates nitric oxide (NO) production. Decreased NO impairs endothelial function leading to an imbalance in microvascular function.

Specifically, we observed inverse associations of microvascular function with PM oxidative potential both in PM_0.18_ and PM_0.18–2.5_ with DTT associations stronger in PM_0.18_ and macrophage ROS associations stronger in PM_0.18–2.5_. Estimated associations of microvascular function with total ROS was stronger than with water-soluble ROS in PM_0.18–2.5_ while no such difference was observed in PM_0.18_. The DTT assay is a chemical (acellular) assay based on the ability of redox-active compounds to transfer electrons from DTT to oxygen [40] while the ROS assay represents cellular production of ROS measured using rat alveolar macrophage cells exposed to particle extracts [19]. The highest oxidative potential measured by both assays were in PM_0.18–2.5_. However, the correlations between the two assays in PM_0.18_ and PM_0.18–2.5_ were weak (R = 0.37 to 0.42), and not correlated in PM_2.5–10_ (−0.11 and −0.20 for DTT with total ROS and water-soluble ROS, respectively). Together with the observation of different associations for DTT and macrophage ROS across the size-fractions, this finding may indicate that these two assays are sensitive to different components of PM and those components lead to health effects that vary depending on particle size distribution and source. Therefore, these two assays complement each other and are informative of the importance of PM under different exposure conditions. Further studies are needed to better understand the relationships between PM components and the associated DTT and macrophage ROS activities.

Few cohort panel studies of within-subject exposure-response relations have examined effects on endothelial function by exposure to ambient air pollution [8, 41–43]. Some [41, 43] but not all [42] found that short-term exposure to PM_2.5,_ sulfate, and BC were associated with FMD of the brachial artery. Most previous studies have investigated the function of forearm conduit arteries. However, the peripheral microvasculature share more similarities in development and anatomy with the microvasculature of the heart than conduit arteries and may be an early cardiovascular risk indicator [44, 45].

A few air pollution studies have evaluated microvascular function. For example, one cross-sectional study of the Framingham Heart Study Offspring Cohorts, Ljungman et al. [7], found no consistent associations between the air pollution exposures, including BC, and microvascular response. However, a cross-sectional study design may not efficiently capture acute air pollution effects given the high temporal and spatial variation in ambient air pollution levels and the variation of outcome and confounders between subjects. Louwies et al. [8] is the only previous study conducted to investigate acute within-subject responses of microvascular function. They examined retinal microvasculature using fundus image analysis in a panel of 84 healthy adults, 22–63 years of age. Consistent with our results, they found that impaired microvascular responses were associated with exposure to BC but with a more acute response (at lag 1 and lag 2 day BC). Many reasons may contribute to the inconsistency among panel studies including: different methods to assess microvascular function, different study population, exposure error, different composition of pollutants, and duration and frequency of exposure assessment. Compared with these above panel studies, our sample size is large, with more repeated measures (*N* = 845), and with more detailed characteristics of the air pollutants.

We found that “protective” effects of O_3_ were confounded by BC, a marker of primary combustion sources. However, the inverse associations between BC and microvascular function persisted after adjusting for O_3_ indicating a robust estimate for BC. This may be attributable partly to meteorological determinants and partly to the observation that high concentrations of a correlated primary pollutant like NO is associated to a reduction of O_3_ [46], both causing the inverse correlation between primary pollutants and O_3_. Therefore, the “protective” effect of O_3_ may be attributed to low levels of primary air pollutants. Another explanation is O_3_ exposure error, given that indoor O_3_ is generally much lower than outdoor concentrations, especially when are windows closed and air conditioning is in use [47].

Unexpectedly, our measurement of personal exposures to NO_x_ was not associated with microvascular function. One explanation may be subject non-compliance, although we have no direct evidence of this. It is possible that the device was not worn as instructed (exposed to outside air and not held in a pocket or purse), or not worn for the 7-day time period requested. In addition, the device is a passive sampler subject to face velocity effects from variations in airflow. Lastly, even though our personal exposure models adjusted for indoor origin of NO_x_, it was possible that this was not fully addressed since the source of adjustment data was from self-administered daily diaries.

We found that microvascular function was significantly inversely associated with OC in PM_2.5–10_, but not in PM_1.8_ or PM_1.8–2.5_. This may because of the strong correlation between OC and inorganic species such as total Cr, Mn, Ni, Cu and Fe (R = 0.74-0.82) in PM_2.5–10_ (Additional file 1: Table S6). These metals had the highest concentrations in PM_2.5–10_ (Additional file 1: Table S3) and they can generate reactive oxygen species by Fenton-type reactions, resulting in adverse health effects [48]. Indeed we found that decreased RHI was significantly associated with Cr, Mn, Ni, Cu and Fe in PM_2.5–10_ with the strongest association observed with Ni (−0.156, 95 % CI:-238, −0.074, Fig. 2). However, co-regression of PM_2.5–10_ OC with PM_2.5–10_ Ni showed that OC was still significantly associated with decreased RHI, and although both exposures showed weaker associations when co-regressed, the confounding was much smaller for OC than for Ni (21 % decrease for OC, 65 % decrease for Ni, Additional file 1: Figure S6). An unmeasured determinant of the OC fraction may be important, perhaps endotoxins. Compared with smaller PM size fractions there is more endotoxin in the coarse than fine PM fractions and consequently greater inflammatory responses by alveolar macrophages [49].

This study enabled us to demonstrate for the first time that associations of impaired microvascular function with primary markers of fossil fuel combustion were stronger for subjects who were former smokers. Given the reduced sample size among subgroups, it is difficult to further explore explanations for the observed effect modification by former smokers. We infer from the present results that former smokers may be more susceptible to exposure to air pollution. It is also possible that being a former smoker is an indicator of other unmeasured co-morbidities or lifestyle habits that place them at increased risk. The finding that subjects who were obese showed stronger associations of impaired microvascular function with air pollutants needs further investigation. However, consistent with our current results, a review by Weichenthal et al. [50] reported that 11 of 14 previous panel studies investigating the health effects of PM_2.5_ on physiological measures of cardiovascular health reported greater adverse responses with exposure to PM_2.5_ among obese adults. It is possible that obesity is associated with enhancement of the proinflammatory effect of air pollution and thus increased susceptibility [51].

Growing evidence [1, 52] supports the view that air pollutants with higher pro-oxidant potential are more capable of generating oxidative stress and inducing inflammation at both respiratory [53, 54] and systemic sites [54] in human population studies. This process may alter the function of the vascular endothelium and initiate endothelial dysfunction. In the present study, we measured the effects of short-term exposure but it is possible that the observed acutely-impaired endothelial function can result in long-term effects following repeated insults as evidenced by recent cohort studies of long-term exposure to traffic-related air pollutants and the development of atherosclerosis [55, 56].

One limitation of our study is that most of our exposure data were obtained from central monitoring stations ranging from 1.58 to 16.37 km to the subject’s residential address. However, any exposure misclassification is likely to be non-differential, leading to an underestimation of the health effect of air pollution [57]. This is validated by our sensitivity analysis in that we did not observe notable changes by restricting the analysis to subjects living within a smaller radius around the air monitoring stations (Additional file 1: Figure S4). Another limitation of our study was that all of the health measures were collected in clinical settings rather than in the home, resulting in non-ambient exposures on the way to the clinic. Further, the lack of data on daily personal exposure prevented us from directly comparing associations with ambient daily data. We also did not assess daily PM composition, which could have provided information on the lag effect of chemical components. Finally, we did not collect information on subjects’ daily diet, which may affect vascular function, and as with any observational study there is the possibility of unmeasured confounding factors in the relationships of interest. Strengths of this study include the repeated measures study design, which enabled control of potentially confounding personal characteristics, the use of non-invasive and relatively technician-independent measures of microvascular function, and the detailed exposure estimation of PM composition and oxidative potential of different particle size-fractions.

## Conclusions

In summary, our results show that microvascular endothelial dysfunction is associated with ambient air pollutants and that these pollutants are linked to primarily mobile sources. Oxidative potential measured both by abiotic DTT and macrophage ROS assays was associated with microvascular dysfunction further highlighting their potential roles in the overall associations between air pollutants and vascular function. European Union National Emission Ceilings and U.S. Environmental Protection Agency-regulated ambient PM_2.5_ mass measurements may not adequately represent risk for cardiovascular disease because they are uncharacterized by composition, source or oxidative potential. Further data are needed using measurements of organic components and oxidative potential across several PM size-fractions and with personal exposures. However, our findings provide clues to the potential mechanisms behind the effects of air pollution on cardiovascular disease and provide further justification of the importance of simultaneously measuring particulate air pollution composition, toxicity and source tracers in assessing adverse cardiovascular health effects.

## Abbreviations

AIC, Akaike’s information criterion; BC, black carbon; BMI, body mass index; CI, confidence interval; CO, carbon monoxide; DTT, dithiothreitol; EC, elemental carbon; FMD, flow-mediated dilatation; HDL, high-density lipoprotein; LDL, low-density lipoprotein; NO_x_, nitrogen oxides; O_3_, ozone; OC, organic carbon; PAHs, polycyclic hydrocarbons; PM_10_, particulate matter with aerodynamic diameter < 10 μm; PM_2.5_, particulate matter with aerodynamic diameter < 2.5 μm; RHI, reactive hyperemia index; ROS, reactive oxygen species; SF-ICPMS, sector field inductively-coupled plasma mass spectrometry; USC, the University of Southern California

## Additional file

Additional file 1: Associations between microvascular function and short-term exposure to traffic-related air pollution and particulate matter oxidative potential supplementary material. (PDF 1.45 mb)
