# Supplementary material for: Associations between microvascular function and short-term exposure to traffic-related air pollution and particulate matter oxidative potential
Source: Environ Health. 2016 Jul 26;15:81. doi: 10.1186/s12940-016-0157-5 (PMC4962442; doi:10.1186/s12940-016-0157-5)
Supplement: Supplementary file 1 — Associations between microvascular function and short-term exposure to traffic-related air pollution and particulate matter oxidative potential supplementary material. (PDF 1.45 mb) [file 12940_2016_157_MOESM1_ESM.pdf]

Supplementary material

Online supplement 1: Missing imputation method for ambient exposure data at SCAQMD

Assuming exposure data were obtained from the nearest air pollution monitoring stations according to the subjects' residential address, then one approach to missing data is to take exposure data from the next nearest monitoring station without missing values (Hansen et al. 2009). However, directly taking missing data from the next nearest monitoring station may introduce a greater systematic exposure error because even though the correlation between the two close stations may be strong, the average exposure concentrations can be quite different (as we found). In addition, it is difficult to compare ambient data obtained from multiple stations to size-fractionated PM concentrations, which were measured only at one station each year. Therefore, for ambient exposure data from the South Coast Air Quality Management District, we utilized data from the two main stations (North Main station for the first year and Anaheim station for the second year) that had the minimal distance to all subjects. Daily exposure data were calculated for each station in the study area from the hourly data, if >75% were available. To impute missing values in the main stations, we first assessed the correlations for each ambient air pollutant between the main stations and other monitoring stations in the study area. Then, if missing data for exposures were observed in the main station, the site used to impute missing data was selected by the highest correlation and by availability of the exposure data. If missing exposure data were not available from any monitoring stations in the study areas and missing values were less than 4 continuous days, then linear interpolation was used to impute missing data. Missing data were imputed using predictions from a simple linear regression model:

$$Y = \beta_0 + \beta_1 X_1 + \varepsilon$$

Where Y is the vector of the predicted variable, β_0 is the estimate of intercept, β_1 is the estimate of slope, X_1 is the exposure variable from closest station, and ε is the error term.

Table S1 shows the missing rate, imputation method, and stations that were used to impute missing data if linear regression was used for each ambient air pollutants. All imputation linear models had $R^2 > 0.8$.

Table S1. Missing rates and results of imputation.

Pollutants	Primary Method	Dependent Variable	Missing Rate	Intercept	Slope	R^2	Secondary Method	Missing Rate	Dependent Variable	Intercept	Slope	R^2	*Δ*mean[b]	*Δ*Std[c]
Downtown Los Angeles n=228														
BC	Linear interpolation[a]		3.95%										3.67%	2.89%
$PM_{2.5}$	Linear interpolation		`2.19%										-0.77%	-1.23%
O3	Linear regression	Burbank	6.14%	2.55	0.79	0.86							-4.82%	-5.20%
NO_X	Linear regression	Burbank	27.19%	2.38	0.91	0.87	Linear regression	8.33%	Pasadena	-8.27	1.99	0.84	-0.49%	-0.71%
CO	Linear regression	Burbank	4.47%	0.23	0.76	0.88							0.30%	-1.01%
Anaheim n=229														
BC	Linear interpolation		2.62%										-0.52%	-1.46%
$PM_{2.5}$	Linear regression	Long Beach	8.30%	4.55	0.79	0.81	Linear interpolation	3.93%					-1.68%	-1.74%
O3	Linear regression	La Habra	10.91%	2.66	1.00	0.90	Linear interpolation	2.18%					-4.52%	-2.14%
NO_X	Linear regression	La Habra	9.61%	0.48	0.97	0.92	Linear interpolation	2.18%					-0.27%	-4.92%
CO	Linear regression	La Habra	4.80%	0.16	0.79	0.87	Linear interpolation	2.18%					3.92%	-0.27%

[a] [a]Linear interpolation was applied where no other available data from nearby stations;
[b]*Δ*mean: change (%) in mean after imputing;
[c]*Δ*Std: change (%) in standard deviation after imputing.

Table S2. Selected organic components measured in $PM_{0.18}$ and $PM_{0.18-2.5}$.

Total polycyclic aromatic hydrocarbons
Fluoranthene
Pyrene
Benzo(ghi)fluoranthene
Benz(a)anthracene
Chrysene
1-Methylchrysene
Retene
Benzo(b)fluoranthene
Benzo(k)fluoranthene
Benzo(e)pyrene
Benzo(a)pyrene
Perylene
Indeno(1,2,3-cd)pyrene
Benzo(g,h,i)perylene
Coronene

Hopanes
17α(H)-22,29,30-Trisnorhopane
17α(H)21β(H)-30-Norhopane
17α(H)21β(H)-hopane
22S-Homohopane
22R-Homohopane
22S-Bishomohopane
22R-Bishomohopane

Selected organic acids
Heptadecanoic acid
Hexadecanoic acid
Octadecanoic acid
Pentadecanoic acid
Tetradecanoic acid
Phthalic acid

Table S3. Descriptive statistics of the selected transition metals.

Metals	N (Missing)	Mean (SD)	IQR	Min	Max
V (ng/m^3)					
$PM_{0.18}$	45 (3)	0.18 (0.08)	0.08	0.07	0.49
$PM_{0.18} – PM_{2.5}$	45 (3)	0.52 (0.26)	0.32	0.18	1.37
$PM_{2.5} – PM_{10}$	44 (4)	0.57 (0.19)	0.16	0.25	1.16
Cr (ng/m^3)					
$PM_{0.18}$	45 (3)	0.31 (0.17)	0.23	0.07	0.72
$PM_{0.18} – PM_{2.5}$	45 (3)	0.46 (0.27)	0.31	0.10	1.28
$PM_{2.5} – PM_{10}$	44 (4)	1.18 (0.53)	0.76	0.30	2.63
Mn (ng/m^3)					
$PM_{0.18}$	45 (3)	0.79 (0.61)	0.75	0.15	2.41
$PM_{0.18} – PM_{2.5}$	45 (3)	1.54 (1.09)	0.92	0.32	5.23
$PM_{2.5} – PM_{10}$	44 (4)	4.77 (1.82)	1.89	1.63	11.35
Ni (ng/m^3)					
$PM_{0.18}$	45 (3)	0.19 (0.1)	0.14	0.03	0.45
$PM_{0.18} – PM_{2.5}$	45 (3)	0.97 (1.2)	0.41	0.19	5.78
$PM_{2.5} – PM_{10}$	44 (4)	0.49 (0.29)	0.46	0.00	1.11
Cu (ng/m^3)					
$PM_{0.18}$	45 (3)	3.21 (2.78)	3.62	0.35	12.09
$PM_{0.18} – PM_{2.5}$	45 (3)	3.86 (2.78)	2.42	0.65	14.19
$PM_{2.5} – PM_{10}$	44 (4)	16.06 (9.28)	12.14	2.46	39.05
Fe (ng/m^3)					
$PM_{0.18}$	45 (3)	47.79 (36.84)	52.21	6.11	140.63
$PM_{0.18} – PM_{2.5}$	45 (3)	64.67 (49.01)	46.46	10.42	217.46
$PM_{2.5} – PM_{10}$	44 (4)	373.50 (159.73)	204.41	107.29	861.48

Abbreviations: IQR: interquartile range; PM: particulate matter.

Table S4. Descriptive statistics of air pollutant measurements by region.

	Los Angeles				Anaheim			
	Mean (SD)	IQR	Min	Max	Mean (SD)	IQR	Min	Max
**Personal Exposures (7-day average)**								
NO_x (ppb)	31.20 (20.60)	20.87	3.99	154.9	27.02 (22.36)	22.45	2.21	160.13
**Ambient Exposures (24-hr Averages)**								
Black Carbon (μg/m^3)	1.58 (0.86)	1.10	0.31	4.08	1.14 (0.84)	0.96	0.13	5.21
$PM_{2.5}$(μg/m^3)	19.17 (9.55)	10.98	3.83	80.58	16.26 (7.77)	7.03	2.68	46.58
CO (ppm)	0.62 (0.25)	0.37	0.11	1.33	0.51 (0.25)	0.28	0.16	1.59
Ozone (ppb)	22.03 (8.03)	12.84	5.22	40.76	21.25 (9.19)	14.14	1.33	43.52
NO_x (ppb)	42.69 (29.05)	34.69	3.60	175.6	33.38 (28.57)	35.04	4.20	142.08
Heat Index (F$^\circ$)	63.84 (9.64)	83.13	42.57	14.85	64.59 (7.89)	84.33	44.79	12.25
**Size-fractionated PM (5-day average)**								
Mass (μg/m^3)								
$PM_{0.18}$	2.25 (0.74)	1.11	1.27	3.68	2.59 (0.97)	1.03	1.17	4.81
$PM_{0.18}$ – $PM_{2.5}$	10.21 (3.21)	4.51	5.39	19.43	6.84 (2.13)	2.57	4.4	13.25
$PM_{2.5}$ – PM_{10}	17.32 (7.5)	9.37	6.92	35.36	12.02 (4.45)	3.46	4.45	25.26
Total PAHs (ng/m^3)								
$PM_{0.18}$	0.37 (0.20)	0.31	0.12	0.77	0.25 (0.23)	0.26	0.03	0.74
$PM_{0.18}$ – $PM_{2.5}$	0.57 (0.47)	0.50	0.07	1.98	0.33 (0.39)	0.37	0.00	1.21
Hopanes (ng/m^3)								
$PM_{0.18}$	0.21 (0.11)	0.17	0.06	0.44	0.12 (0.12)	0.10	0.01	0.39
$PM_{0.18}$ – $PM_{2.5}$	0.29 (0.21)	0.36	0.08	0.82	0.12 (0.15)	0.19	0.00	0.48
OA (μg/m^3)								
$PM_{0.18}$	28.01 (9.69)	15.09	12.85	46.14	24.06 (11.90)	11.90	9.64	57.98
$PM_{0.18}$ – $PM_{2.5}$	21.58 (16.41)	18.36	8.31	61.43	14.17 (15.32)	15.32	0.56	43.22
OC (μg/m^3)								
$PM_{0.18}$	1.05 (0.34)	0.5	0.47	1.69	1.34 (0.4)	0.45	0.80	2.35
$PM_{0.18}$ – $PM_{2.5}$	1.71 (0.68)	0.97	0.91	3.48	1.35 (0.86)	1.16	0.53	3.48
$PM_{2.5}$ – PM_{10}	0.77 (0.19)	0.28	0.35	1.06	0.55 (0.19)	0.23	0.28	1.12
EC (μg/m^3)								
$PM_{0.18}$	0.29 (0.10)	0.13	0.17	0.55	0.23 (0.15)	0.13	0.09	0.60
$PM_{0.18}$ – $PM_{2.5}$	0.24 (0.10)	0.16	0.12	0.45	0.05 (0.04)	0.03	0.01	0.13
$PM_{2.5}$ – PM_{10}	0.05 (0.03)	0.04	0.00	0.09	0.02 (0.02)	0.03	0.00	0.06
Total ROS (μg Zym/m^3)[a]								
$PM_{0.18}$	21.98 (12.90)	21.15	3.9	53.2	17.3 (11.15)	12.8	2.1	46.7
$PM_{0.18}$ – $PM_{2.5}$	196.68 (91.54)	135.4	39.7	394	68.13 (28.4)	29.5	26.2	132.2
$PM_{2.5}$ – PM_{10}	92.15 (47.20)	77.55	15.6	181	21.83 (7.75)	12.95	8.7	33.1
Water-soluble ROS (μg Zym/m^3)								
$PM_{0.18}$	19.12 (12.44)	17.95	3.8	50.6	14.03 (9.89)	10.8	1.50	39.6
$PM_{0.18}$ – $PM_{2.5}$	165.69 (93.85)	122.1	34.8	421.9	61.46 (26.29)	19.4	18.6	131.4
$PM_{2.5}$ – PM_{10}	37.02 (24.58)	41.6	2.4	84.2	10.66 (5.39)	7.60	3.40	22.5
Dithiothreitol (nmol/min/m^3)								
$PM_{0.18}$	0.1 (0.05)	0.06	0.04	0.24	0.09 (0.05)	0.06	0.02	0.21
$PM_{0.18}$ – $PM_{2.5}$	0.24 (0.07)	0.08	0.12	0.39	0.28 (0.10)	0.10	0.12	0.48
$PM_{2.5}$ – PM_{10}	0.3 (0.12)	0.17	0.13	0.53	0.17 (0.05)	0.04	0.10	0.29
Total metals[b] ((ng/m^3)								
$PM_{0.18}$	47.79 (31.96)	46.17	7.22	116.70	57.84 (47.14)	78.91	12.05	153.39
$PM_{0.18}$ – $PM_{2.5}$	75.59 (51.43)	30.39	25.76	237.10	67.94 (55.44)	63.14	12.89	203.44
$PM_{2.5}$ – PM_{10}	478.31 (122.92)	139.10	246.60	729.30	298.49 (171.63)	97.58	112.00	914.57

Abbreviations: CO: carbon monoxide; EC: elemental carbon; IQR: interquartile range; OC: organic carbon; PAHs: polycyclic aromatic hydrocarbons; PM: particulate matter; ROS: Reactive oxygen species;

[a]Zym: μg Zymosan equivalent units;

[b]Total sum of transition metals include V, Cr, Mn, Ni, Cu and Fe.

Table S5. Spearman correlation matrix of ambient, personal air pollutants and heat index by region

	Los Angeles						Anaheim					
	BC	CO	NO_X	$PM_{2.5}$	O_3	Heat Index (F∘)	BC	CO	NO_X	$PM_{2.5}$	O_3	Heat Index (F∘)
24-hr averages of ambient exposures												
BC (μg/m^3)		0.88	0.90	0.23	-0.63	-0.13		0.85	0.91	0.47	-0.76	-0.45
CO (ppm)			0.91	0.12	-0.68	-0.31			0.87	0.46	-0.78	-0.32
NO_x (ppb)				0.17	-0.69	-0.20				0.25	-0.85	-0.46
$PM_{2.5}$(μg/m^3)					0.02	0.26					-0.17	-0.15
O_3 (ppb)						0.56						0.54
7-day averages of personal exposure [a]												
Personal NOx (ppb)	0.25	0.24	0.26	-0.07	-0.21	-0.06	0.66	0.63	0.66	0.30	-0.64	-0.44

Abbreviations: BC: black carbon; CO: carbon monoxide;
[a]Correlations for personal NOx were calculated with 7-day average of ambient pollutants and heat index.

Table S6. Spearman correlations of selected PM components and transition metals in three size-fractions

	OC	EC	T ROS	WS ROS	DTT	OC	EC	T ROS	WS ROS	DTT	OC	EC	T ROS	WS ROS	DTT
**$PM_{0.18}$**															
V	0.15	0.02	0.25	0.24	0.24	-0.19	-0.28	-0.20	-0.13	0.05	-0.31	-0.40	-0.27	-0.19	-0.37
Cr	0.41	0.49	0.17	0.16	0.48	0.45	0.18	0.16	0.12	0.23	0.36	0.19	-0.12	-0.34	0.30
Mn	0.74	0.60	0.25	0.25	0.79	0.57	0.16	0.02	0.05	0.41	0.34	0.25	-0.32	-0.54	0.31
Ni	0.21	0.44	0.34	0.29	0.28	0.26	0.23	0.26	0.14	0.03	0.21	0.10	-0.01	-0.13	0.07
Cu	0.60	0.69	0.24	0.23	0.71	0.73	0.35	0.29	0.32	0.48	0.50	0.43	-0.02	-0.30	0.55
Fe	0.76	0.63	0.28	0.27	0.81	0.58	0.18	0.03	0.04	0.42	0.34	0.26	-0.32	-0.54	0.31
**$PM_{0.18-2.5}$**															
V	-0.54	-0.17	0.07	0.06	-0.42	-0.25	0.07	0.27	0.31	-0.26	-0.29	-0.20	0.36	0.55	-0.15
Cr	0.34	0.73	0.28	0.27	0.56	0.67	0.51	0.51	0.50	0.43	0.46	0.38	0.31	0.11	0.47
Mn	0.56	0.73	0.24	0.24	0.70	0.68	0.37	0.34	0.33	0.55	0.48	0.33	0.09	-0.11	0.39
Ni	-0.21	0.22	0.17	0.15	-0.18	0.23	0.34	0.53	0.55	0.12	0.08	0.15	0.52	0.54	0.29
Cu	0.46	0.76	0.20	0.20	0.64	0.73	0.53	0.48	0.47	0.50	0.56	0.46	0.23	-0.01	0.55
Fe	0.56	0.69	0.21	0.22	0.69	0.66	0.38	0.31	0.30	0.51	0.50	0.37	0.07	-0.14	0.43
**$PM_{2.5-10}$**															
V	-0.30	0.24	0.24	0.22	-0.15	0.06	0.38	0.43	0.46	0.09	0.35	0.24	0.60	0.63	0.06
Cr	0.15	0.74	0.24	0.25	0.38	0.70	0.75	0.62	0.65	0.31	0.82	0.71	0.58	0.32	0.69
Mn	0.07	0.67	0.22	0.23	0.30	0.62	0.74	0.58	0.59	0.30	0.81	0.68	0.61	0.39	0.64
Ni	-0.08	0.62	0.25	0.26	0.21	0.53	0.76	0.67	0.69	0.15	0.74	0.63	0.70	0.47	0.59
Cu	0.30	0.83	0.18	0.21	0.50	0.83	0.76	0.61	0.61	0.32	0.81	0.75	0.46	0.18	0.72
Fe	0.22	0.78	0.24	0.26	0.41	0.74	0.75	0.58	0.60	0.34	0.85	0.74	0.54	0.28	0.68

Abbreviations: DTT: dithiothreitol; EC: elemental carbon; OC: organic carbon; PM: particulate matter; T: total; ROS: reactive oxygen species; WS: water-soluble.

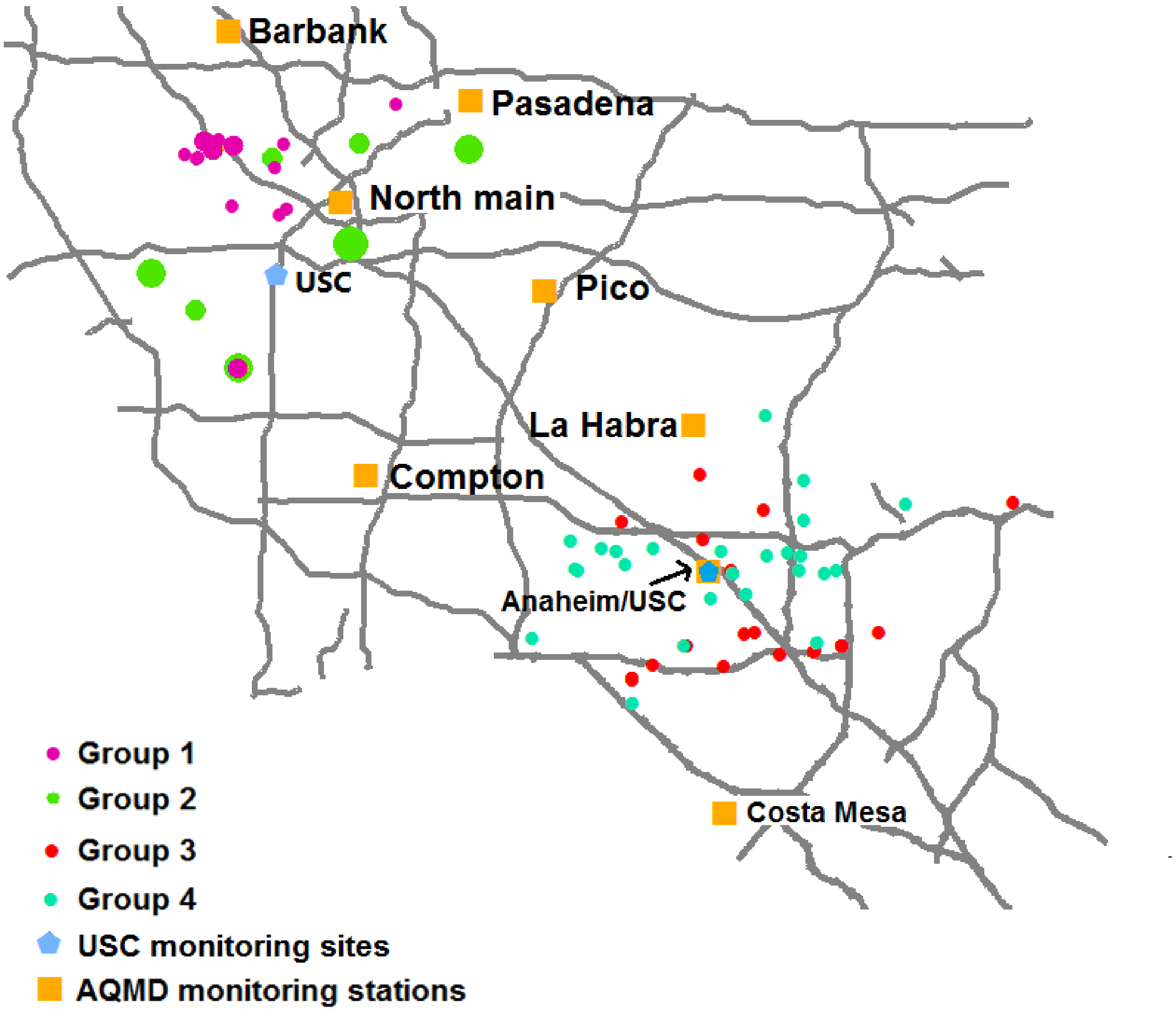

Figure S1. Geographic locations of central monitoring stations and subjects' residential addresses in the study area. Size of circles reflect number of subjects.

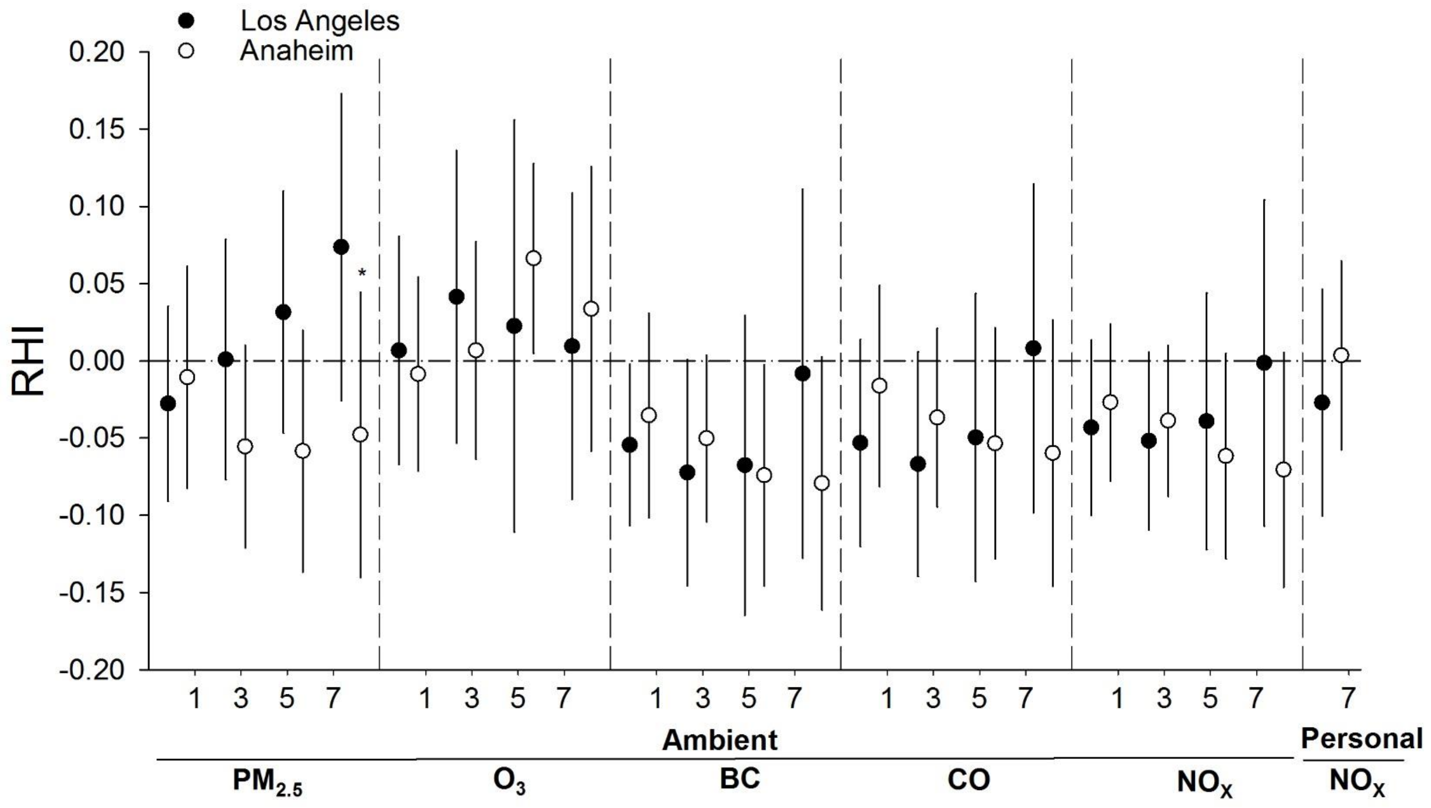

Figure S2. Association of microvascular function with a one interquartile range increase of air pollutants by study region. RHI: reactive hyperemia index. Numbers on X axis refer to the exposure averaging time: 1-day, 3-day, 5-day and 7-day. *p < 0.1, compared with no effect modification by obesity status. BC: black carbon.

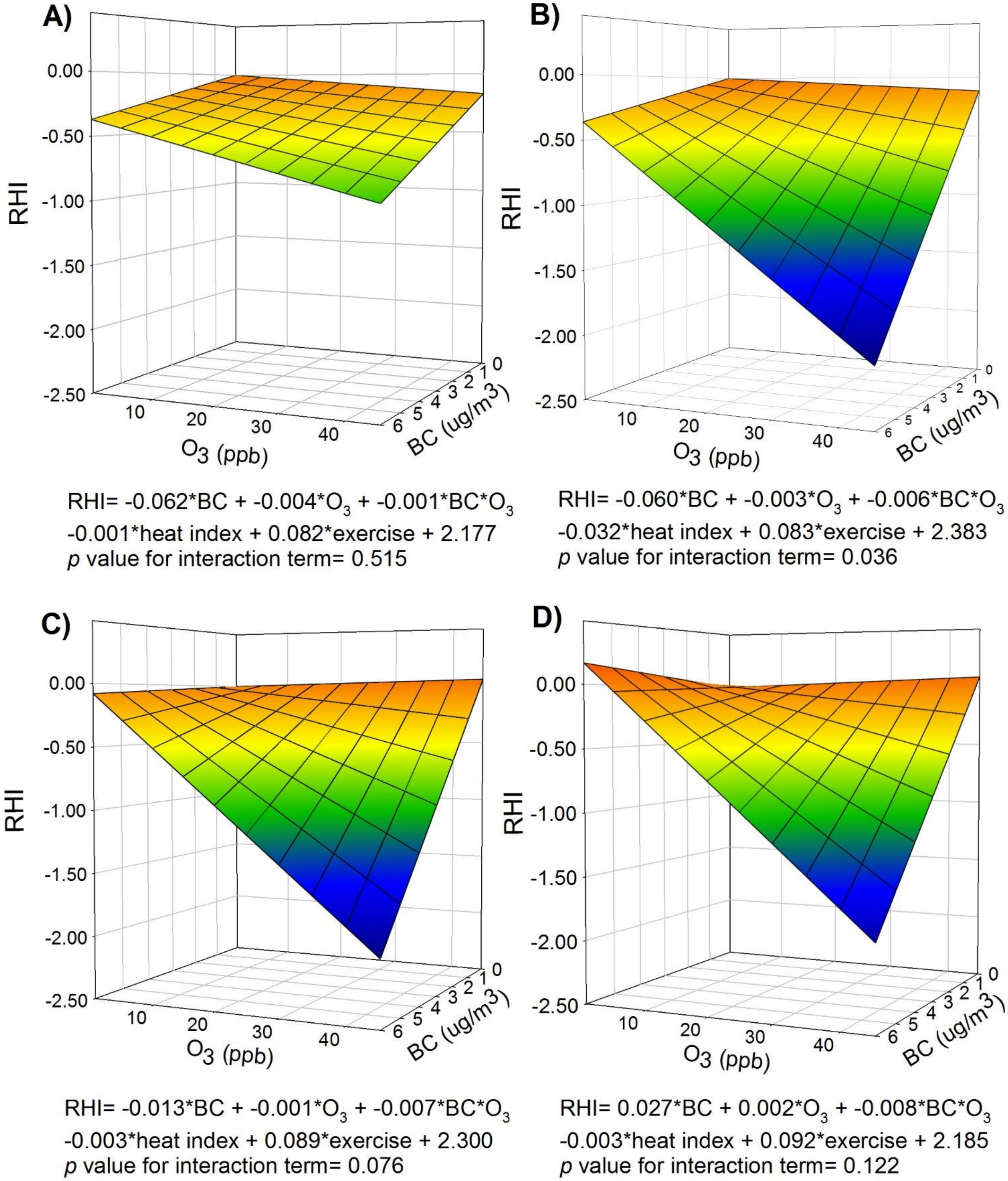

Figure S3. Relation of microvascular function to interaction between black carbon and O_3. Changes in reactive hyperemia index (RHI) per unit increase of BC, O_3 and their interaction across 1 day (A), 3 days (B), 5 days (C), and 7 days (D) averaged before each subject's measurement. BC: black carbon;

Ln(FeNO)= -0.0076*BC + -0.0063*O_3 + 0.0022*BC*O3 + -0.0008*heat index + 3.2979

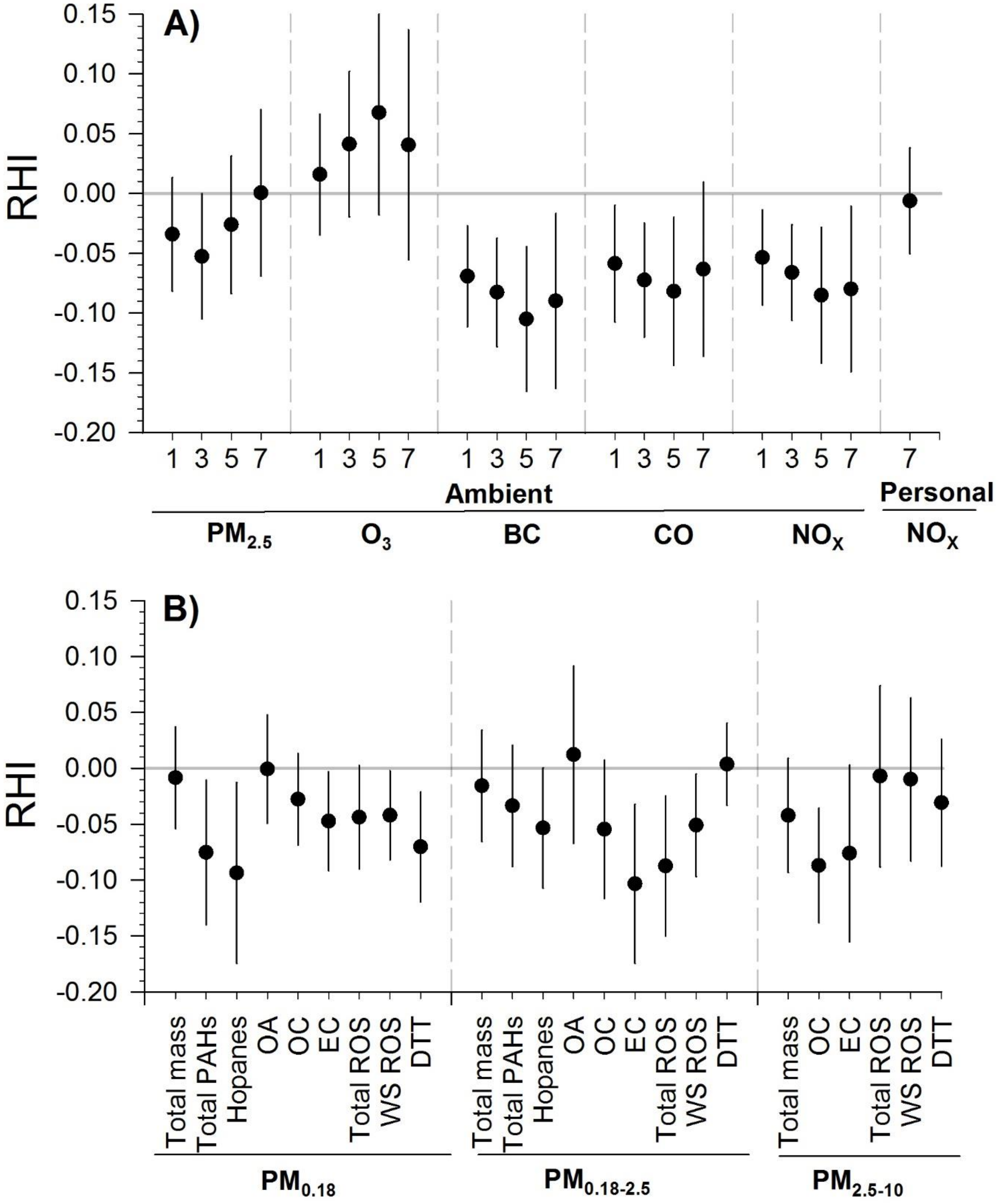

Figure S4. Sensitivity analysis of relations between microvascular function and air pollution restricted to subjects living within the 90th percentile of subjects' residential distance to the stations. Association of reactive hyperemia index (RHI) with a one interquartile range increase of ambient and personal air pollutants Exposures were averaged across 1 day, 3 days, 5 days, and 7 days preceding each subject's measurement with sensitivity analysis restricted to subjects living within 13.75 km (A); and the PM components in three different size-fractions for exposure averages across 5 days preceding each subject's RHI measurement with sensitivity analysis restricted to subjects living within 11.41 km (B). BC: black carbon; DTT: dithiothreitol; EC: elemental carbon; OA: Organic acids; OC: organic carbon; PAH: polycyclic aromatic hydrocarbons; ROS: reactive oxygen species; WS: water-soluble.

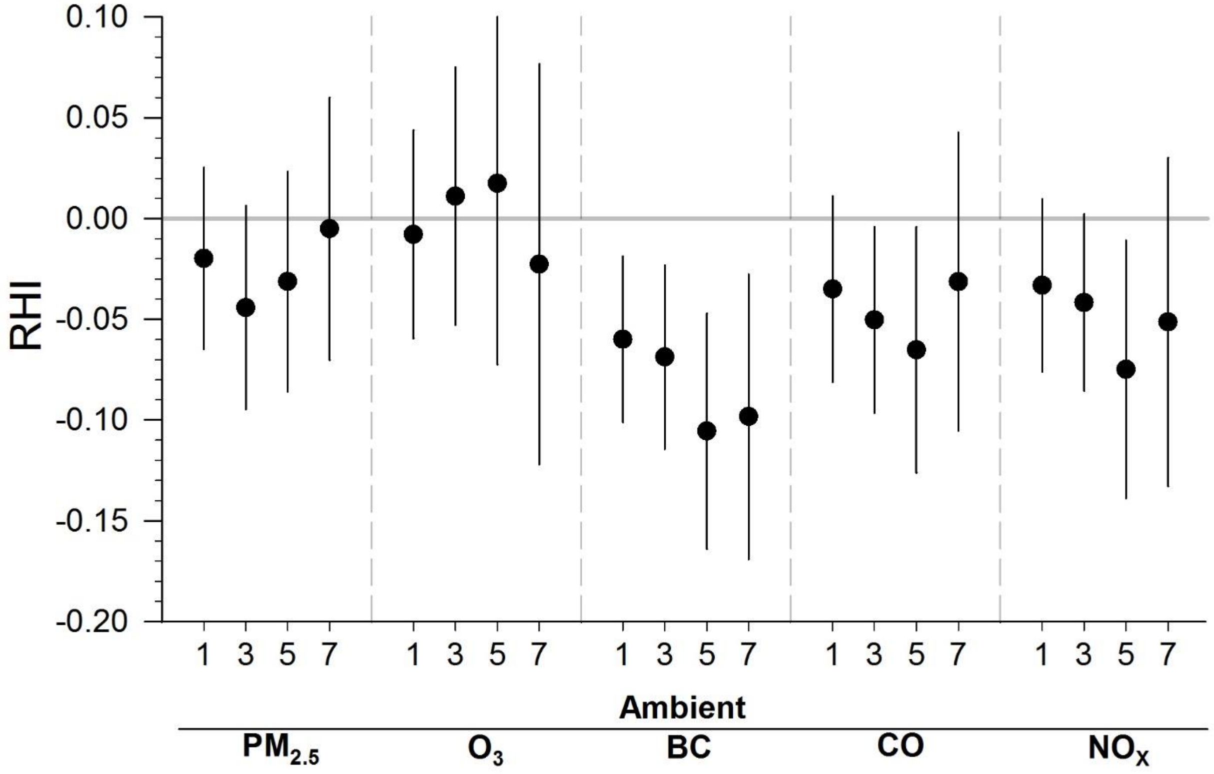

Figure S5. Sensitivity analysis of relations between microvascular function and air pollution excluding imputed exposure values. Association of reactive hyperemia index (RHI) with a one interquartile range increase of ambient and personal air pollutants for exposures averaged across 1 day, 3 days, 5 days, and 7 days preceding each subject's measurement. BC: black carbon.

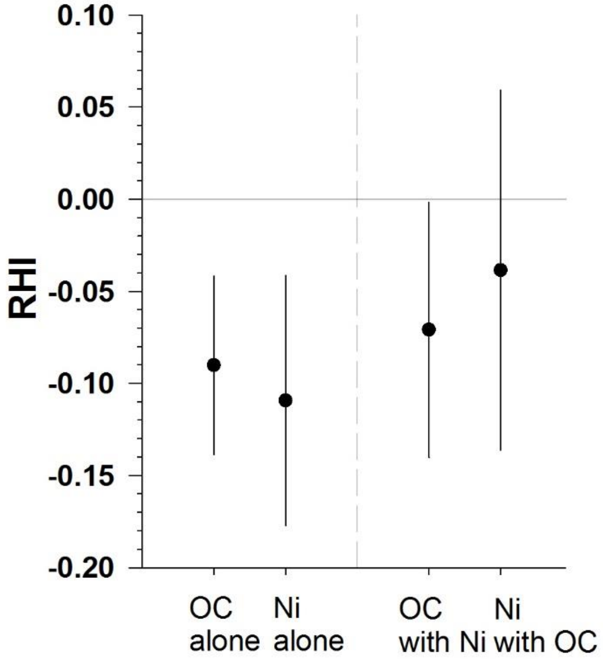

Figure S6. Association of microvascular function with a one interquartile range increase of OC co-regressed with Ni in $PM_{2.5-10}$. Air pollutants are averaged across 5 days preceding each subject's measurement of reactive hyperemia index (RHI). OC: organic carbon.
